# Effects of food neophobia and oral health on the nutritional status of community-dwelling older adults

**DOI:** 10.1186/s12877-022-03013-7

**Published:** 2022-04-18

**Authors:** Takako Yodogawa, Yasuhito Nerome, Junya Tokunaga, Hiromichi Hatano, Mika Marutani

**Affiliations:** 1 Department of Oral Health Science, Faculty of Nursing and Social Welfare, Kyushu University of Nursing and Social Welfare, 888, Tomio Tamana-shi, Kumamoto, 865-0062 Japan; 2 Faculty of Medicine School of Health Sciences, Kagoshima University, 8-35-1, Sakuragaoka Kagoshima-shi, Kagoshima, 890-8544 Japan; 3 Department of Nursing, Faculty of Health Science, Aino University, 4-5-4, Higashi-Ohda, Ibaraki-city, Osaka 567-0012 Japan; 4 National Institute of Public Health, 2-3-6 Minami, Wako-shi, Saitama, 351-0197 Japan

**Keywords:** Food neophobia, Malnutrition, Oral-health–related QOL, Oral function

## Abstract

**Background:**

Food preferences and oral health of older adults greatly affect their nutritional intake, and old-age–related increase in food neophobia may consequently reduce food intake in older adults. This study aimed to determine the impact of food neophobia and oral health on nutritional risk in community-dwelling older adults.

**Methods:**

This cross-sectional study included 238 independent adults aged ≥ 65 years (mean, 76.3 ± 7.3 years). The survey items included a Food Neophobia Scale, frequency of protein intake, oral-health–related quality of life (QOL) assessment, and oral diadochokinesis (ODK; /pa/, /ta/, /ka/) as an index of oral function. Nutritional status was assessed using the Mini Nutritional Assessment®, and based on a cutoff value of 24 points, respondents were categorized as well-nourished (≥ 24 points, Group 1) or at risk of malnutrition (< 24 points, Group 2). A logistic regression model was used to calculate the adjusted odds ratio (adj-OR) with 95% confidence interval (CI) to identify risks factors for malnutrition associated with food neophobia and oral health.

**Results:**

Factors associated with the risk of malnutrition in the older population were higher food neophobia (adj-OR = 1.036, 95% CI: 1.007–1.067) and lower oral function (OR = 0.992, 95% CI: 0.985–0.999) and lower oral-health–related QOL (adj-OR = 0.963, 95% CI: 0.929–0.999).

**Conclusions:**

Older adults at risk of developing malnutrition may have higher food neophobia and lower oral function and oral-health–related QOL. Factors contributing to preventing malnutrition include predicting the risk of malnutrition based on the oral health indicators that older people are aware of, signs appearing in the oral cavity, minor deterioration, and providing dietary guidance about food neophobia. Notably, these approaches represent novel strategies for nutrition support that can be implemented based on a multifaceted understanding of the eating habits of older adults.

## Background

In 2007, Japan became a super-aged society, as defined by the World Health Organization and the United Nations, with an aging rate of over 21%. The speed of this event remains unparalleled and this rate is the highest worldwide. Hence, low nutrition among older adults is becoming more apparent, and the number of older adults at risk of developing malnutrition is expected to increase with the increasing aging rate. In particular, according to a survey conducted in 2014, 17.8% of those aged ≥ 65 years were prone to malnutrition, and this figure increased to approximately 20% among those aged ≥ 80 years [1]. Further, about one-third to one-quarter of the general older population is thought to be malnourished [2]. Among older adults, nutritional status is closely related to general health, disease development, and quality of life (QOL), making it an extremely important issue [3, 4]. Notably, this trend of low nutrition in older adults appears to be occurring on a global scale [5, 6] and thus requires urgent intervention.

Dietary practices of older adults are shaped by long-term eating habits that begin in youth and develop throughout life, and as it has been reported that older adults tend to have less diverse diets, there is concern that this population may have a narrowed range of preferences and monotonous food intake [7]. Birch et al. [8] attempted to explain the idea of food neophobia, which is defined as a concept that captures the tendency to hesitate and avoid novel foods, as a factor that influences food intake and nonconsumption. Interestingly, food neophobia has also been observed in other omnivores [9]. Remarkably, food neophobia has been shown to be more prevalent in older age groups [10, 11], and a study by Tuorila et al. reported higher levels of food neophobia in older adults aged > 66 years compared with other age groups [12]. Further, a study by Heikki et al. [13] reported that those with high food neophobia had lower levels of nutrients, such as proteins. This is especially important in older adults as it may lead to the development of diseases. Nevertheless, a relationship between food neophobia and low nutrition has not been established yet.

 Intuitively, healthy functioning of the oral cavity is essential for the physical functioning of older adults as it ensures adequate nutrition. Although it has been shown that a reduction in the number of teeth [14] and a decline in oral function [15] and oral-health–related QOL [16] are associated with nutritional status, previous studies have only illustrated the independent impact of each factor. In clinical practice, despite the potential problem of food neophobia, oral support and nutritional guidance are provided without a clear understanding of the food recognition and acceptance process [17]. Therefore, the purpose of this study was to introduce food neophobia and oral health as a model and to identify factors that are strongly related to nutritional risk.

## Methods

### Study design and sample

This cross-sectional study was conducted between 2015 and 2016 and included 238 community-dwelling individuals aged ≥ 65 years. Representatives of all the senior citizens’ association groups that collaborated with the health center in a rural area of Kumamoto prefecture were requested to explain the purpose of the survey and provide cooperation. To increase the representativeness of the older adult population, we selected participants equally from groups clustered according to the region. Convenience sampling was used during the survey. A preliminary survey conducted in the same area among older adults (*n* = 20; age = 78.7 ± 6.4 years) indicated no major problems with the survey methodology or questionnaire responses. The main survey was conducted by visiting the community activity site of a senior citizens’ association group that had obtained consent from the participants. The dental hygienist in-charge of the survey, who had been trained in advance, directly asked all participating senior citizens to cooperate in the survey. An investigator distributed self-administered questionnaires to the participants who provided consent and checked for omissions. Oral function was examined based on oral diadochokinesis (ODK) using an oral function measurement device, and this assessment was performed directly after the Mini Nutritional Assessment® (MNA) interview and the measurement of calf and mid-arm circumferences. The study participants were independent older adults who were living at home, did not suffer from dementia (confirmed by a physician), and were able to walk independently.

### Survey items

Information on basic attributes, food neophobia and intake, protein intake frequency, oral-health–related QOL, and nutritional status (MNA) was collected from the study participants. The number of teeth and oral function were also assessed.

#### Demographic data and medical history of the study participants

Information on the participants’ age, sex, underlying diseases, family structure, individual responsible for cooking, and health guidance knowledge on physical and oral health was collected.

#### Food neophobia scale

We used the Food Neophobia Scale developed by Pliner et al. (1992), the reliability of which has been previously confirmed [18]. The scores on the Food Neophobia Scale ranged from 10 to 70, with higher scores indicating a greater level of food neophobia. The scale comprised 10 items, and the options for answers ranged from 1: “I don’t think so at all” to 7: “I absolutely think so” on a 7-point scale.

#### Frequency of protein intake

The Dietary Variety Score (DVS) [19] was used in this study to assess the frequency of protein intake by summing the scores for meat, seafood, eggs, soy products, and milk as follows: 1: “Almost every day,” 2: “Once every two days,” 3: “Once a week,” and 4: “I hardly eat.” The scores for each question item were reversed so that higher scores indicated greater frequency of protein intake. The total score obtained for the five foods ranged from 5 to 20, with higher scores indicating more frequent protein intake.

#### Oral-health–related QOL

We used the oral health impact profile-14 (OHIP14) (14-item version) [20] to measure oral-health–related QOL. The options ranged from 1: “never” to 5: “always” for each factor, and as the scores for each item were reversed, the total scores ranged from 7 to 70, with higher scores indicating a higher QOL.

#### Nutritional status

Nutritional status was evaluated using the MNA [21, 22], which comprises 18 items. The participants were classified into the following three categories depending on their scores: well-nourished (24–30 points), at risk of malnutrition (17–23.5 points), and malnourished (0–16.5 points). The MNA ranged from 0 to 30 points, with lower scores indicating a higher risk of malnutrition. This is a well-validated tool and this method of scoring yielded a sensitivity of 96%, specificity of 98%, and positive predictive value of 97% [23].

#### Oral function

Oral function was evaluated using ODK, in which the number of times /Pa/, /Ta/, and /Ka/ were pronounced in succession for 5 s was measured using an oral function measurement device (Kenkokun Handy, Takei Scientific Instruments Co. Ltd.) [24]. Motor function of the lip was assessed using the sound /Pa/, that of the anterior region of the tongue was assessed using /Ta/, and that of the posterior region of the tongue was assessed using /Ka/.

### Ethical considerations

The following aspects were explained to all potential participants: aims and outline of the study, participant rights (voluntary participation and cooperation and right to refuse participation), protection of privacy by questionnaire coding, protection of personal information and confidentiality of data, and destruction of the questionnaire immediately after study completion. Only those who gave their consent were included in the study. This study was approved by the ethical review board of the Kagoshima University Faculty of Medicine (approval number 292).

### Statistical analysis

Demographic data and medical history variables were evaluated using descriptive statistics. Based on a cutoff value of 24 for the MNA score, participants were categorized as well-nourished (> 24 points, Group-1) or at risk of malnutrition (< 24 points, Group-2). The two groups were compared using Mann–Whitney *U* test and χ^2^ test. A logistic regression model was used to calculate adjusted odds ratios (adj-OR) with 95% confidence interval (CI) for risk factors of malnutrition that were associated with food neophobia and oral health. The multivariable logistic regression analysis was performed with the forced substitution of age and sex as well as the stepwise selection of food neophobia, protein intake frequency, ODK, number of teeth, and oral-health–related QOL. A two-sided *p-*value of < 0.05 was considered statistically significant. Statistical analyses were performed using SPSS ver.25.0 (IBM).

## Results

The basic attributes of the 238 participants are shown in Table 1. The study population included 67 (28.2%) men and 171 (71.8%) women.

**Table 1:** Basic attributes (*n* = 238)

Variable		Statistic values
Age, mean (SD)		76.33 (7.39)
Sex, frequency (%)	Male	67 (28.2)
	Female	171 (71.8)
Family composition, frequency (%)	Living alone	89 (37.4)
	Married couple only	90 (37.8)
	Other living with family	59 (24.8)
Underlying condition, frequency (%)	None	47 (19.7)
	Cardiovascular disease	100 (42.0)
	Diabetes	24 (10.1)
	Bone fracture	22 (9.2)
	Gastrointestinal disease	23 (9.7)
	Cerebrovascular disease	17 (7.1)
	Pneumonia	16 (6.7)
	Respiratory disease	14 (5.9)
	Other	41 (17.2)
Food neophobia, mean (SD)		38.91 (11.78)
Oral-health–related QOL (OHIP14), mean (SD)		60.92 (8.52)
Frequency of protein intake mean (SD)	Total	14.70 (2.7)
	Fish	3.04 (0.77)
	Meat	2.71 (0.75)
	Eggs	2.82 (0.96)
	Milk	2.82 (1.14)
	Soybeans	3.29 (0.80)
Nutritional status (MNA®)		
mean (SD)		25.32 (3.49)
frequency (%)	Well-nourished	173 (72.68)
	At risk of malnutrition	64 (26.89)
	Malnutrition	1 (0.004)
Oral function [oral diadochokinesis (ODK)]		
mean (SD)	ODK_pa (/10 sec)	55.50 (9.61)
	ODK_ta (/10 sec)	50.96 (9.87)
	ODK_ka (/10 sec)	49.75 (10.55)
Number of teeth, mean (SD)		15.02 (10.87)
Dentures, frequency (%)	Yes	148 (62.2)
	No	90 (37.8)

Table 2 compares nutritional status as a function of key variables between Groups 1 and 2, revealing significant differences between them in terms of age (*p* = 0.001), sex distribution (*p* = 0.018), food neophobia (*p* = 0.001), oral-health–related QOL (*p* = 0.024), ODK (*p* = 0.001), and number of teeth at present (*p* = 0.024). However, there were no obvious differences in the frequency of protein intake.

**Table 2:** Comparison of nutritional status as a function of major variables and measures (*n* = 238)

		Group 1 well-nourished (*n* = 173)		Group 2 risk of malnutrition (*n* = 65)		*U* value	*p*-value
Age, mean (SD)		74.94 (6.63)		80.03 (8.07)		3515.0	0.001
Sex ^a^, n (%)	Male	56 (32.4%)		11 (16.9%)			0.018
	Female	117 (67.6%)		54 (83.0%)			
Food neophobia, mean (SD)		43 (2.74)		37 (11.82)		3881.5	0.001
Oral-health–related QOL, mean (SD)		61.84 (7.96)		58.46 (9.50)		4564.5	0.024
Frequency of protein intake, mean (SD)		14.83 (2.74)		14.35 (2.57)		7258.0	0.279
Oral diadochokinesis (ODK), mean (SD)		157.55 (35.82)		116.10 (71.58)		3587.0	0.001
Number of teeth, mean (SD)		16.08 (10.76)		12.22 (10.74)		4561.0	0.024

Mann–Whitney *U* Test

^a^*χ*^2^ test

Table 3 lists adj-ORs for the risk factors of malnutrition listed in Table 2. The results of the χ^2^ test for the model were *p* < 0.05, and each variable was significant. The results of the Hosmer–Lemeshow test were *p* = 0.27, and the model fit well with a discriminant accuracy rate of 78.2%. Participants with higher age or greater food neophobia had a higher risk of malnutrition, with an adj-OR of 1.036 (95% CI: 1.007–1.067, *p* = 0.016) for food neophobia. Conversely, participants with higher oral-health–related QOL or ODK had a lower risk of malnutrition, with an adj-OR of 0.963 (95% CI: 0.929–0.999, *p* = 0.044) for oral-health–related QOL and an adj-OR of 0.992 (95% CI: 0.985–0.999, *p* = 0.036) for ODK.

**Table 3:** Odds ratios and 95% confidence intervals for risk factors of malnutrition associated with food neophobia and its covariates

	B value	OR	Adjusted 95% CI	*p*-value
Age	0.050	1.051	0.998–1.107	0.059
Sex (male, 1; female, 0)	−0.606	0.546	0.252–1.181	0.124
Food neophobia	0.036	1.036	1.007–1.067	0.016
Oral diadochokinesis (ODK)	−0.008	0.992	0.985–0.999	0.036
Oral-health–related QOL	−0.037	0.963	0.929–0.999	0.044

Multivariable logistic regression model with adjustment for all variables is shown in Table 2.

## Discussion

This cross-sectional study was conducted on 238 community-dwelling older adults. Food neophobia and oral health were introduced as models, and their association with nutritional risk was examined. The results showed that food neophobia, oral function, and oral-health–related QOL were associated with a risk of malnutrition. According to the 2015 census, the aging rate of people aged ≥ 65 years in the study area was approximately 31.2%, which was higher than the national average of approximately 26.7% in Japan. Interviews were conducted to increase the reliability of the data obtained from the participating older adults, and as the survey targeted older adults who voluntarily participate in community activities, we thought that this group would be highly interested in diet and health [25, 26]. The MNA revealed 0.4% of the population to be malnourished, 26.89% to be at risk of malnutrition, and 72.68% to be well-nourished, indicating that the nutritional status of the study population was similar to that reported in previous studies [27]. For ODK, although the obtained values tended to be lower than the standard values for independent older people [24], as similar values have been reported in previous studies [28], oral function was not considered to be compromised in our study population compared with other study populations.

One study reported a 25% decrease in daily calorie consumption from the age of 40 years to 70 years [29]. Among older adults, energy intake is reduced due to lower energy expenditure, and in general, older adults tend to lose weight [30] if physiological factors associated with aging lead to anorexia, which can affect their nutritional status. The results of this study showed that age was not significantly associated with the risk of malnutrition.

### Effect of food neophobia on nutritional status

Food neophobia in older adults was found to be significantly associated with the risk of malnutrition and has been reported to significantly reduce the intake of 20 nutrients [31], attesting to the high external validity of this study. A decrease in the intake of three or more nutrients has been suggested to be significantly associated with frailty that is independent of energy intake [32], implying that food restriction due to food neophobia may be a mechanistic cause of malnutrition.

In contrast, familial practices of food neophobia have been shown to be highly influenced by vertical inheritance and by family members who live together [33–35]. In particular, it has been reported that when consuming novel foods, an individual’s appetite decreases if the person they are eating with shows disgust but increases if the other person displays pleasure [36]. Additionally, verbal and nonverbal cues for communication and sharing, including negative evaluation of novel foods in meal situations, have been shown to influence food acceptance [37]. Thus, it can be inferred that the evaluation of a meal and communication cues as well as the background of the relationship among the family members or others who provide the meal act as key factors that influence the intake of novel foods. Ensuring adequate food intake and nutritional status in older adults also requires interventions that not only directly address food neophobia but also factors that may indirectly control the effects of food neophobia. In fact, Birch et al. suggested that repeated exposure to unfamiliar foods can lead to a change in familiarity, and that more frequent opportunities to do so represent a strategy to reduce the effects of food neophobia [8, 38]. In older adults with food neophobia, providing health information on novel foods may increase food preference [39]. As food intake and nonintake are affected by a complex interplay of innate human nature and individual experiences, it is necessary to understand the level of individual food neophobia to provide specific and adequate support for effective intervention methods. Apart from the acceptance of novel foods, even known foods may be perceived as unfamiliar due to differences in cooking methods and appearance, and these factors may lead to reluctance and lower motivation for consumption. In contrast, when older adults with food neophobia consume foods that they perceive as novel, the risk of malnutrition may decrease as innovative ways of providing meals become available.

### Effects of oral health on nutritional status

ODK was found to be significantly associated with the risk of malnutrition, suggesting that decreased lip and tongue motor function and dexterity may reduce the ability to ingest food and swallow, consequently affecting nutritional status. It has also been shown that it is important to evaluate oral function when assessing nutritional status. Oral frailty is the process by which the accumulation of minor deterioration in oral conditions, such as teeth, oral function, and oral hygiene, associated with aging increases oral vulnerability and leads to eating dysfunction. Oral frailty is considered to be one of the precursors or accelerators of generalized frailty, and it has been reported that those who suffer from oral frailty have a 2.4-fold higher risk of developing physical frailty [40]. Oral frailty is thought to be a transition from a decline in oral function to nutritional impairment and the need for nursing care. As signs of oral frailty have been observed in individuals in their 50s, such as early symptoms of xerostomia and poor oral hygiene [41], it is necessary to start addressing oral frailty from a younger age. Importantly, prevention may be possible by encouraging the incorporation of inexpensive autonomous training into the activities of daily life [42–44], and it might be possible to avoid the progression from poor oral function to malnutrition by detecting signs of trivial changes related to the oral cavity and by providing appropriate intervention at an early stage.

Oral-health–related QOL was found to be significantly associated with the risk of malnutrition. Gil-Montoya et al. [45] reported an association between high oral-health–related QOL and adequate nutritional status (MNA), and our results corroborate these findings.

A decline in the masticatory and swallowing functions in older adults leads to a decline in oral health that the individual is aware of because of restrictions in the quality and quantity of food. A relationship between the decline in chewing and swallowing abilities and nutritional status has been reported [46], and it has been pointed out that xerostomia and poor oral hygiene, which are observed in older adults, can reduce gustatory effects [47]. Furthermore, it has been shown that impaired taste is closely related to appetite [48] and that anorexia leads to malnutrition [49]. Thus, complex problems in the oral cavity that individuals are aware of can lead to a poor oral-health–related QOL and affect the nutritional status.

The association between the number of teeth at present and oral-health–related QOL has been reported in many studies [50, 51], and tooth loss is generally associated with nutritional deficiencies [14]. In the present study, there was no association between the number of teeth at present and nutritional status (MNA), indicating that an objective assessment of oral health, such as the number of teeth, does not always coincide with oral health awareness. This aspect not only emphasizes the importance of determining an individual’s perceived oral health status (functional, social, and psychological aspects) when assessing nutritional status but also highlights that the use of oral-health–related QOL, along with clinical parameters, may be useful in predicting nutritional risk in the older population.

### Limitations of the study and future research

The generalizability of causal relationships in nutritional status require careful evaluation and longitudinal studies for an in-depth understanding. However, we used a cross-sectional study design to easily evaluate the nutritional status of a group of older adults living in a wide area.

In addition, this study revealed no association between protein intake and nutritional status. However, considering the level of nutrient intake required for older adults, protein deficiency tends to result in malnutrition; therefore, it is necessary to consider improving the accuracy of collecting data regarding ingested foods using dietary recording and photography methods [52]. In future research, the examination of reliable survey methods should be performed while ensuring the reliability of the method targeting community-dwelling older adults.

Dietary intake is closely related to oral function, and oral-function–related QOL is believed to have a wide range of effects on direct feeding behavior as well as nutritional risk. In the future, it is necessary to consider specific methods for maintaining oral health to reduce nutritional risk. Concomitantly, it is necessary to verify the effective contents and embodiments of guidance on dietary habits and oral health based on the evaluation of food preferences, as in this study.

The proportion of older adults who are responsible for their own cooking is influenced by their level of care and age, and the transition to a diet that is dependent on others has been described [53], wherein it has been reported that those who rely on family members, food delivery services, and older adult care facilities for food preparation dislike greater number of foods and, hence, are at risk of malnutrition [54]. This indicates that the nutritional status of older adults is dependent on the relationship with the cook, and the quality of communication while serving meals has been reported to affect the nutritional quality of older adults [55]. It can be inferred that the empirical evaluation of factors related to food preferences, intake, and nutritional status of those who depend on cooking will be possible by simultaneously evaluating the relationship with the cook and various aspects of communication.

## Conclusions

Our results suggest that older adults at risk of developing malnutrition have greater food neophobia and lower oral function and oral-health–related QOL. This indicates the importance of determining older adults’ perceived oral health status, predicting the risk of malnutrition based on the observation of oral signs and modest functional decline, and providing dietary guidance that considers food neophobia. Such an approach could lead to the implementation of new strategies for nutrition programs in the community and clinical settings based on a multifaceted understanding of eating in the older population.

## Data Availability

Datasets used and analyzed during the current study are available from the corresponding author on reasonable request.

## Abbreviations

MNA: Mini Nutritional Assessment
OHIP: Oral health impact profile
ODK: Oral diadochokinesis
CI: Confidence interval
QOL: Quality of life
