# Correction: Effects of food neophobia and oral health on the nutritional status of community-dwelling older adults

**DOI:** 10.1186/s12877-022-03097-1

**Published:** 2022-05-17

**Authors:** Takako Yodogawa, Yasuhito Nerome, Junya Tokunaga, Hiromichi Hatano, Miki Marutani

**Affiliations:** 1 Department of Oral Health Science, Faculty of Nursing and Social Welfare, Kyushu University of Nursing and Social Welfare, 888, Tomio, Tamana-shi, 865-0062 Kumamoto, Japan; 2 Faculty of Medicine School of Health Sciences, Kagoshima University, 8-35-1, Sakuragaoka, Kagoshima-shi, 890-8544 Kagoshima, Japan; 3 Department of Nursing, Faculty of Health Science, Aino University, 4-5-4, Higashi-Ohda, Osaka 567-0012 Ibaraki-city, Japan; 4 National Institute of Public Health, 2-3-6 Minami, Wako-shi, 351-0197 Saitama, Japan

**Correction to: BMC Geriatr 22, 334 (2022)**

**https://doi.org/10.1186/s12877-022-03013-7**

After publication of this article [1], the authors reported that in this article the author name Miki Marutani was incorrectly written as Mika Marutani.

The original article [1] has been updated.
